# Assessment of finger motor function that reflects the severity of cognitive function

**DOI:** 10.20407/fmj.2020-013

**Published:** 2020-12-16

**Authors:** Shota Suzumura, Yoshikiyo Kanada, Aiko Osawa, Junpei Sugioka, Natsumi Maeda, Taishi Nagahama, Kenta Shiramoto, Katsumi Kuno, Shiori Kizuka, Yuko Sano, Tomohiko Mizuguchi, Akihiko Kandori, Izumi Kondo

**Affiliations:** 1 Department of Rehabilitation Medicine, National Center for Geriatrics and Gerontology, Obu, Aichi, Japan; 2 Faculty of Rehabilitation, School of Health Sciences, Fujita Health University, Toyoake, Aichi, Japan; 3 Center for Technology Innovation – Artificial Intelligence, Research and Development Group, Hitachi Ltd., Kokubunji, Tokyo, Japan; 4 Optronics Innovation Dept., Optronics Division, Maxell, Ltd., Yokohama, Kanagawa, Japan; 5 Center for Exploratory Research, Research & Development Group, Hitachi Ltd., Kokubunji, Tokyo, Japan

**Keywords:** Cognitive function, Finger tapping, Dexterity, Mini-Mental State Examination (MMSE)

## Abstract

**Objectives:**

We conducted a finger tapping movement test using a finger tapping device with magnetic sensors (UB-2) and performed multiple regression analyses using a number of finger movements parameters to estimate the severity of cognitive impairment.

**Methods:**

The subjects of this study were 64 patients, including 44 diagnosed with Alzheimer’s disease (AD) (mean age: 73.8±7.0 years) and 20 diagnosed with mild cognitive impairment (MCI) (mean age: 76.7±4.2 years). For the finger-tapping movement tasks, we tested single-hand (left and right) tapping, simultaneous tapping of both hands, and alternate tapping between hands. After measurement, multiple regression analysis adjusted for age and sex was performed to predict the Mini-Mental State Examination (MMSE) score from the calculated hand parameters.

**Results:**

Relatively high standardized partial regression coefficients were observed for the following two parameters: standard deviation (SD) of distance rate of velocity peak in extending movement and the SD of contact duration. The coefficients of determination (R^2^) ranged between 0.1 to 0.28.

**Conclusions:**

Our results suggest the possibility that these parameters may be used to assess cognitive function. We shall obtain large-scale data from older people to examine the possibility of these parameters to be used as an early diagnostic tool for dementia patients.

## Introduction

The World Health Organization estimated that in 2015, there were 47 million people with dementia worldwide, and predicted that the number would triple by 2050 due to the aging of the population.^1^ In Japan, the number of dementia patients is increasing and is currently approximately 6.3 million. When combining this with the number of individuals with mild cognitive impairment (MCI), which is estimated to be about 4 million, about one out of four older people aged 65 or higher is believed to have dementia or preliminary dementia, which has become a major social problem.^2^

The Lancet International Commission on Dementia Prevention, Intervention, and Care has indicated the importance of dementia prevention and has reported that the prevalence of dementia could be reduced by half if dementia onset is delayed for five years.^3^ Although it is not currently possible to suppress pathological brain changes of dementia patients, it would be important to detect the risk of dementia at an early stage to prevent its onset and progression.

Alzheimer’s disease (AD), which accounts for about 60% of all dementia, presents with episodic memory disorder as an early symptom and follows a chronically progressive course, accompanying other cognitive disorders.^4,5^ On the other hand, MCI is defined as an intermediate state between normal cognition and dementia and is associated with a preclinical stage of AD.^6^ Recent reports state that dementia in its early and pre-symptomatic stages including MCI, present not only with memory disorder but also motor^7–9^ and sensory dysfunction.^10,11^ Therefore, it is considered important to comprehensively examine these functional aspects of the brain. We focused on finger movements (finger tapping) as one of the measures and hypothesized that finger movements may exhibit subtle abnormalities associated with pathological changes in the brain before the appearance of initial symptoms of dementia. To prove the hypothesis, we proceeded with our preliminary study on finger tapping movements in dementia patients using a finger movement measurement device (UB-1; Finger tapping device with magnetic sensors, Hitachi Computer Peripherals Co. Ltd., Kanagawa, Japan) as a tool to evaluate motor dysfunction in dementia patients easily in a short time.^12^ The results indicated the possibility that finger dexterity declines already at MCI, and identified parameters that decline in parallel with the severity of cognitive dysfunction. Previous studies on finger function in dementia patients reported declined fine motor control and dexterity of the fingers and fewer finger tapping movements.^13–15^ A decrease in finger tapping velocity was also reported by Muller in mild AD patients^16^ and Ott in mild to moderate AD patients,^17^ respectively.

Studies on finger dexterity demonstrated the decline with age, and the difference between sexes was also noted.^18–20^ Although, as described above, the hand function of dementia patients has been studied, there have been no reports examining the relationship between cognitive function and hand function of dementia patients in multivariable models.

**Figure 1:**
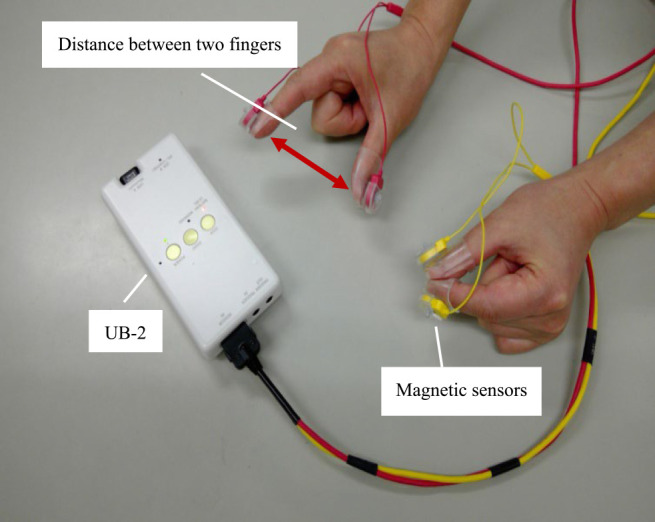
Finger Tapping Device with Magnetic Sensors (UB-2) The yellow cable is attached to the left thumb and index finger, and the red cable is attached to the right thumb and index finger. The main body of the device is connected to a PC, and the wave patterns of the finger tapping can be checked on the PC. It is lighter than UB-1 and is easy to carry.

Therefore, the purpose of this study was to measure finger tapping movements using UB-2 (finger tapping device with magnetic sensors, Maxell Holdings, Ltd, Tokyo, Japan, Figure 1), which is an improved version of the magnetic sensor-type finger-tapping device described above, and to perform multiple regression analysis on finger tapping movements that reflect the severity of cognitive function.

## Methods

### Subjects

The subjects of this study included patients diagnosed with either AD or MCI at the Center for Comprehensive Care and Research on Memory Disorders at the National Center for Geriatrics and Gerontology. MCI patients were included in this study to have a wide range of subjects to improve the prediction accuracy of finger motor skills that reflect the severity of cognitive function. The diagnostic criteria for AD and MCI were in accordance with the criteria specified by the National Institute on Aging-Alzheimer’s Association (NIA/AA)^21^ and by Petersen,^22^ respectively. The subjects were 69 patients, including 49 AD patients (mean age: 73.4±6.7 years) and 20 MCI patients (mean age: 76.7±4.2 years). The exclusion criteria were disturbances in consciousness, higher brain dysfunction, such as aphasia and apraxia, apparent paralysis, and sensory impairment due to stroke, dexterity disorder, and tremor.

### Ethical consideration

All study subjects or their families were given sufficient verbal and written explanations of the purpose of this study, and only those who gave consent were included in the study. This study was approved by the Ethics and Conflicts of Interest Committee of the National Center for Geriatrics and Gerontology (Approval number 623-7).

### Filling in the medical questionnaire sheet and assessment of cognitive function

Before undergoing finger tapping measurement, patients with AD or MCI received a structured interview to confirm their name, age, sex, medical history, and dominant hand, and the background information related to dementia, using a questionnaire sheet (Table 1). We also performed the Mini-Mental State Examination (MMSE), which can quantitatively evaluate cognitive function.

### Measurement method

**Figure 2:**
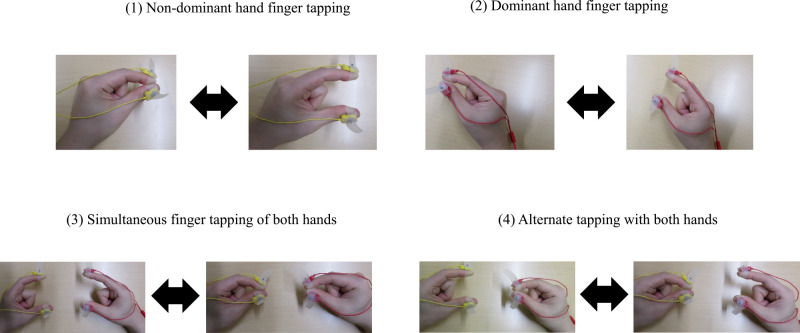
Finger-tapping movement (1) Non-dominant hand finger tapping: Perform finger tapping with the left thumb and index finger as fast as possible. (2) Dominant hand finger tapping: Perform finger tapping with the right thumb and index finger as fast as possible. (3) Simultaneous finger tapping of both hands: Perform finger tapping of both hands as fast as possible at the same time. (4) Alternate tapping with both hands: Perform finger tapping alternately with the left and right hands, as fast as possible. Perform (1) to (4) described above for 15 s, keeping the distance between the thumb and index finger 3 to 4 cm.

**Figure 3:**
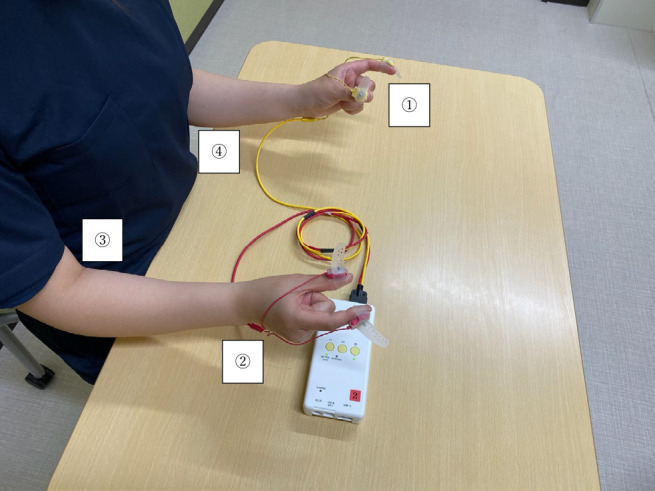
Points to note when measuring ①: Rest the third to fifth fingers lightly in the palm. ②: Keep the wrist joints in slight dorsiflexion. ③: Keep the forearms in the intermediate position between pronation-supination, and the upper arms close to the body. ④: Keep the elbow joint off the desk

We used a finger tapping device with magnetic sensors (UB-2, Maxell Holdings, Ltd, Tokyo, Japan) for measurement (Figure 1). The size of the device was 69×28×140.5 mm (width×depth×height), the weight was 210 g, and the built-in battery provided 3.5 h of continuous use when fully charged. We performed the measurement with the subjects sitting in a chair in a quiet environment. The measurement method was as follows: The yellow and red cables shown in Figure 1 were attached to the left and right fingers, respectively. The cables were attached to the dorsal side (nail side) of the thumb and the index fingertips of the respective hand. The subjects were then instructed to perform finger tapping movements (repetitive tapping by extension and flexion of the index finger against the thumb tip). The finger tapping task consisted of four types of movements: tapping of a single hand (left or right hand), simultaneously with both hands (left and right tapping at the same time), and alternate hands (alternate left and right-hand tapping) (Figure 2). When measuring, we paid attention that the subject kept the following positions: (1) the elbow joint was off the desk, (2) the forearms were in the intermediate position between pronation-supination, and the upper arms were kept close to the body, (3) the wrist joints were in slight dorsiflexion, and (4) the third to fifth fingers rested lightly in the palm (Figure 3). Before measuring each movement, the subjects were instructed to practice once for approximately five seconds to confirm the degree of understanding of the tapping task. We asked them to tap as fast as possible. After practice, the subjects performed each task in the order of left hand, right hand, simultaneous, and alternate hands, and the movements were measured for 15 seconds.

These four tasks were selected in this study for the following reasons: (1) There is a significant difference between the dominant and non-dominant hand, and (2) it is important to assess the dependency (simultaneous movements of both hands) and independence (alternate hands) when measuring both hands.

### Statistical methods

To predict the MMSE score from finger parameters, we performed a multiple regression analysis adjusted for age and sex. The parameters used in the multiple regression analysis were determined using the stepwise variable selection method. The initial model was a constant term alone, and the F value for the criterion for the input of variables was 0.05, and the criterion for exclusion was 0.10. After the finger tapping measurement, 44 parameters were calculated using the accompanying software, Just Tap.^12^ However, in this study, parameters with similar meanings were omitted to avoid problematic multicollinearity in applying multiple regression analysis, and the 36 parameters shown in Table 2 were used. The statistical significance level was set at *p*<0.05, and SPSS Statistics ver. 26.0 was used as the analysis software.

## Results

Based on the inclusion and exclusion criteria, 64 dementia patients (44 AD patients and 20 MCI patients) were included in the study. All subjects were right-handed. Among the patients with AD, five patients were excluded from the study, including one with a history of stroke, two with a history of cervical spondylosis, one diagnosed with corticobasal degeneration, and one who had difficulty with daily living activities due to tremors. The subjects’ characteristics are shown in Table 3.

### A case of wave patterns in an elderly individual and an AD patient

**Figure 4:**
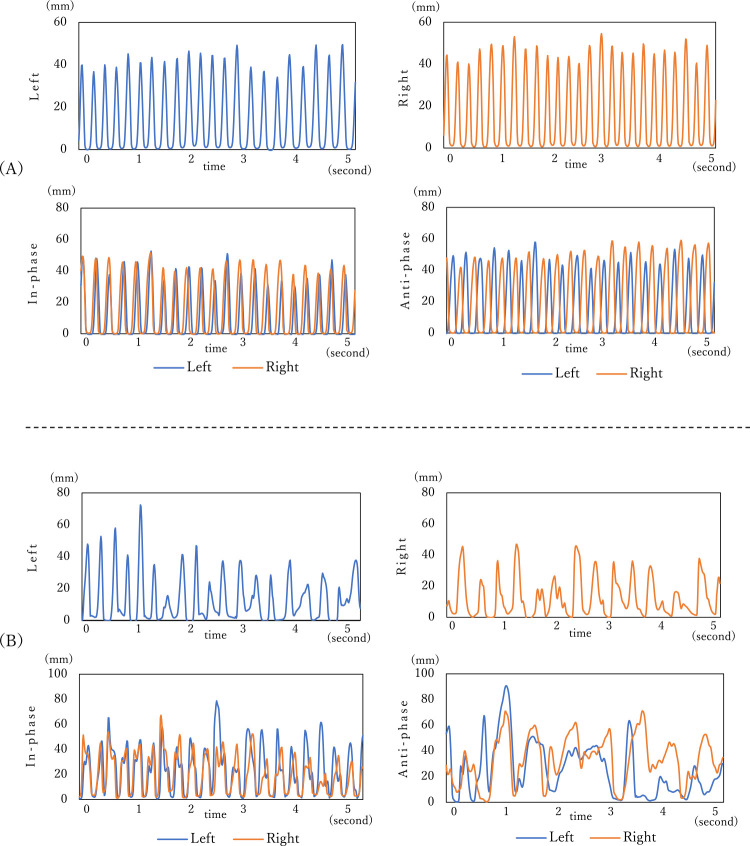
Wave patterns for an elderly individual and an AD patient (A) A male of advanced age in his seventies (B) A female AD patient in her seventies

Figure 4 shows the measured wave patterns. (A) is the measurement result for a male in his seventies, who is a family member of a patient and accompanies the patient to our center, leads an independent social life, and has never visited our center for memory disorder. (B) is the measurement result for a female AD patient in her seventies, who is one of the subjects of this study. The wave pattern for (A) shows frequent and constant finger-tapping movements. However, the wave pattern for (B) shows that the tapping number was less and the tapping pattern was not constant.

### Multiple regression analysis using the MMSE score as the dependent variable

Table 4 shows the results of a multiple regression analysis using the MMSE score as the dependent variable adjusted for age and sex. The “standardized partial regression coefficient,” which indicates the strength of the relative association of the independent variable with the dependent variable, was the largest in the order of the standard deviation (SD) of distance rate of velocity peak in extending movement, (Table 2, No. 17), the SD of contact duration (Table 2, No. 25), the SD of inter-tapping interval (Table 2, No. 29), average of distance rate of velocity peak in extending movement (Table 2. No. 14), and the slope of approximate line of local maximum points (Table 2, No. 5). No significant differences were seen in other parameters. In addition, regarding the relationship between each task, the following had a standardized partial regression coefficient of 0.3 or larger (or –0.3 or smaller): The SD of distance rate of velocity peak in extending movement (Table 2, No. 17) between single-hand tapping (right hand) and simultaneous tapping of both hands, the SD of contact duration (Table 2, No. 25) between single-hand tapping (left-hand) and left hand in simultaneous tapping of both hands, and the SD of inter-tapping interval (Table 2, No. 29) between the right hand and the left hand in alternate hand tapping. The coefficients of determination (R^2^) were 0.1 to 0.28.

## Discussion

In this study, we performed multiple regression analyses adjusted for age and sex to predict the MMSE scores using finger parameters. The standardized partial regression coefficient tended to be high for the SD of distance rate of velocity peak in extending movement (No. 17) and the SD of the contact duration (No. 25). The SD of contact duration was reported to be indicative of the relationship between the MMSE score and finger function in patients with dementia.^23^ The standard partial regression coefficient for the SD of contact duration tended to be high in our study, indicating that the parameters related to contact time are highly likely to be one of the parameters significantly associated with the cognitive function of patients with dementia. In addition, since the standard partial regression coefficient of the SD of distance rate of velocity peak in extending movement (No. 17) tended to be high, it was suggested that those with low MMSE scores may have an inconsistent distance to reach maximum speed, leading to an unstable finger extension movement. Therefore, the results of this study indicate that it is highly likely that the SD of contact duration (No. 25) is a useful parameter to evaluate variability when fingers are in contact with each other, whereas the SD of distance rate of velocity peak in extending movement (No. 17) is a useful parameter to evaluate variability when fingers are not in contact with each other.

Regarding the relationship among the tasks, the SD of distance rate of velocity peak in extending movement (No. 17) was significantly different between single-hand finger tapping (right hand) and right-hand finger tapping during simultaneous both-hand tapping. A significant difference was also seen in the SD of contact duration (No. 25) between single-hand finger tapping (left hand) and left-hand finger tapping during simultaneous both-hand tapping. Between right- and left-hand tapping in the alternate hand task, a significant difference was seen in the SD of inter-tapping interval (No. 29). Our results supported the findings of a previous study that demonstrated that the contact time of single-hand (left) tapping and left-hand tapping in simultaneous hand tapping was significantly longer in patients with AD or MCI than in healthy elderly people.^23^ Dementia patients have also shown more declined finger function compared to healthy elderly individuals when performing tasks that require coordination of both hands, such as alternate hands, and rhythm.^12^ A significant difference was found in the SD of inter-tapping interval (No. 29) of the alternate hand task, which is a parameter related to rhythm in our study. In patients with dementia, atrophy of not only the cerebral cortex but also the basal ganglia^24^ and corpus callosum,^25^ which are involved in the coordination of the left and right hands, was observed. The two-hand alteration task was more difficult compared to single-hand and simultaneous both-hand tasks, as it required moving both hands independently, and was, therefore, more likely to show a difference in finger function.

Progression rate from MCI to AD is believed to be around 4% to 10% per year,^26^ and it has been reported that 50% of MCI progresses to AD in five years.^27^ However, a definite conclusion about the progression time has not yet been reached. Early diagnosis of AD has become possible, to a certain degree, based on biomarkers in cerebrospinal fluid, such as Aβ, total tau, and phosphorylated tau^28,29^, morphological abnormalities of the brain shown on Magnetic Resonance Imaging (MRI),^30^ and abnormal distribution of cerebral blood flow detected by Single Photon Emission Computerized Tomography (SPECT)^31^ and Positron Emission Tomography (PET).^32^ However, diagnostic imaging based on these advanced technologies has challenges, such as financial and physical burdens on the subject and the time required for measurement and analysis. Compared to these diagnostic modalities, finger tapping measurement is considered to have a lesser burden on the subjects for the following reasons: (1) Introduction to the test is easy, (2) measurement can be performed on those with cognitive impairment as it only requires limited movement of the fingers, and (3) the measurement can be completed in a short time (approximately 5 min). If it becomes possible to estimate the cognitive function of dementia patients to some extent by finger tapping measurement, which will advance the research on finger function, it may contribute to early diagnosis.

This study has several limitations. First, although one of the factors that affect dexterity is potentially coexisting extrapyramidal disorder,^33,34^ we did not evaluate its influence in this study. Second, we judged the level of cognitive function only by the MMSE. Third, the Edinburgh Handedness Inventory^35^ is generally used to assess the dominance of a person’s hand, but we judged dominance based on the questionnaire alone, in consideration of the subject’s fatigue. It is necessary to comprehensively evaluate cognitive function not only by the MMSE, but also by assessing attention and executive function, and to judge hand dominance using a handedness test in the future.

In this study, we measured finger tapping movements using a finger-tapping device with magnetic sensors. We examined the finger movements that reflected the severity of cognitive function using finger movement quantification parameters, whose results of using quantification parameters of finger movements suggested that the SD of distance rate of velocity peak in extending movement (No. 17) and the SD of contact duration (No. 25) were the parameters significantly associated with cognitive function. We aim to use finger tapping movements for one of the screening evaluation items in the future. For that aim, we are planning to collect more data targeting people with advanced age in a large-scale group, such as at a health class in the area. We believe that it would be possible to conduct the finger tapping measurement to detect motor disorders associated with dementia easily in a short time if we can select one task instead of conducting four tasks, including single-hand (left- and right-hand) tasks and simultaneous and alternate two-hand tasks.

## Supplementary Material

PDF-Japanese

## Figures and Tables

**Table1:** Medical questionnaire sheet

		ID	
Name		Serial number	
Birthday		Gender	
Dominant Hand	Right・Left	Age	

No	Question	Answer
1	Have you been diagnosed with kidney disease, liver disease, or heart disease?	( Yes・No )
2	Have you been diagnosed with mental disease, neurological disease, or muscle disease?	( Yes・No )
3	Have you been diagnosed with cervical spondylosis?	( Yes・No )
4	Have you had a stroke (cerebral hemorrhage, cerebral infarction, subarachnoid hemorrhage)?	( Yes・No )
5	Are you under treatment for high pressure?/Is you maximal blood pressure higher than 160 mmHg?	( Yes・No )
6	Are you under treatment for diabetes?	( Yes・No )
7	Are you taking medicine? If yes, Drug name（ ）	( Yes・No )
8	Do you find it difficult to move your limbs or do they become numb?	( Yes・No )
9	Do you have tremor?	( Yes・No )
10	Do you feel that it interferes with activities of daily living?	( Yes・No )
11	Do you have difficulty in swallowing?	( Yes・No )
12	Do you find it difficult to put on and take off your clothes and to attach buttons?	( Yes・No )
13	Do you find it difficult to use chopsticks?	( Yes・No )
14	Is a pacemaker or deep brain stimlation (DBS) attached?	( Yes・No )
15	Please tell us about other diseases you have had in the past ( )	
16	Please tell us about other diseases you are currently undergoing treatment for ( )	

**Table2:** List of finger parameters

1 Max of distance amplitude 2 Total traveling distance 3 Avg. of local max. distance 4 SD of local max. distance 5 Slope of approximate line of local max. points 6 SD of local max. distance in three adjacent taps 7 Max. of velocity amplitude 8 Avg. of local max. velocity 9 Avg. of local min. velocity 10 SD of local max. velocity 11 SD of local min. velocity 12 Energy balance 13 Number of freezing calculated from velocity 14 Avg. of distance rate of velocity peak in extending movement 15 Avg. of distance rate of velocity peak in flexing movement 16 Ratio of distance rates of velocity peak in extending and flexing movements 17 SD of distance rate of velocity peak in extending movement 18 SD of distance rate of velocity peak in flexing movement	19 Max of acceleration amplitude 20 Avg. of local max. acceleration in extending movement 21 Avg. of local min. acceleration in extending movement 22 Avg. of local max. acceleration in flexing movement 23 Avg. of local min. acceleration in flexing movement 24 Avg. of contact duration 25 SD of contact duration 26 Number of zero crossover points of acceleration 27 Number of freezing calculated from acceleration 28 Avg. of tapping interval 29 SD of inter-tapping interval 30 Inter-tapping interval variability 31 Skewness of inter-tapping interval distribution 32 SD of inter-tapping interval in three adjacent taps 33 Avg. of phase difference between the left hand and right hand tapping 34 SD of phase difference between the left hand and right hand tapping 35 Similarity of hands 36 Time lag of similarity of hands

Max: Maximum; Min: Minimum; Ave: Average; SD: Standard deviation Standard deviation and coefficient of variation are parameters with high similarity to each other and indicate “variation.” In this study, to avoid multicollinearity, we used standard deviation parameters and omitted parameters indicating the coefficient of variation.

**Table3:** Characteristics of the subjects

Characteristic	AD group (N=44)	MCI group (N=20)
Age±SD (years)	73.8±7.0	76.7±4.2
Gender (%)	Men 19 (43)	Men 11 (55)
	Women 25 (57)	Women 9 (45)
Education±SD	10.9±2.5	12.4±3.5
Dominant hand	All cases right-handed	All cases right-handed
MMSE±SD (/30 points)	19.1±5.9	24.8±2.9
Barthel Index±SD (/100 points)	94.3±10.3	99.3±1.4

AD: Alzheimer’s disease; MCI: Mild Cognitive Impairment; SD: standard deviation

**Table4:** Results of multiple regression analysis with MMSE score as the dependent variable

Task	Hand	Independent variables	Standardized coefficients β	95% CI		p-value
				lower	upper	
Dominant hand task	Right	SD of distance rate of velocity peak in extending movements	–0.442	–169.469	–54.291	0.000
		**R^2^**	**0.2**			
Non-dominant hand task	Left	SD of contact duration	–0.449	–188.290	–63.294	0.000
		Avg. of distance rate of velocity peak in extending movement	–0.243	–39.909	–1.697	0.033
		**R^2^**	**0.28**			
In-phase task	Right	SD of distance rate of velocity peak in extending movements	–0.466	–152.075	–53.079	0.000
		**R^2^**	**0.22**			
	Left	SD of contact duration	–0.320	–224.048	–34.450	0.008
		Slope of approximate line of local max. points	–0.237	–2.334	–0.011	0.048
		**R^2^**	**0.19**			
Anti-phase task	Right	SD of inter-tapping interval	–0.319	–37.371	–5.253	0.01
		**R^2^**	**0.1**			
	Left	SD of inter-tapping interval	–0.323	–44.477	–6.513	0.009
		**R^2^**	**0.104**			

MMSE: Mini-Mental State Examination; CI: confidence interval; R^2^: coefficient of determination; Max: Maximum; Ave: Average; SD: Standard deviation There was no significant difference in the other parameters. * SD of distance rate of velocity peak in extending movement (No. 17): variability in the values calculated as the ratio of the position at the maximum velocity of the finger extension to the amplitude. * SD of contact duration (No. 25): variability of the duration while two fingers (the thumb and index finger) contact each other. * SD of inter-tapping interval (No. 29): variability of inter-tapping interval (the difference in time of the minimum points of two consecutive tapping movements). The larger this value, the more inconsistent the inter-tapping interval. * Average distance rate of velocity peak in extending movement (No. 14): The mean value calculated as the ratio to the amplitude of the distance at the maximum velocity during finger extension. * Slope of approximate line of local max. points (No. 5): The slope of the linear regression of the relationship between the maximum points (the maximum point per tapping movement) and the duration.
