# Supplementary material for: Assessment of finger motor function that reflects the severity of cognitive function
Source: Fujita Med J. 2020 Dec 16;7(4):122–9. doi: 10.20407/fmj.2020-013 (PMC8761821; doi:10.20407/fmj.2020-013)
Supplement: Supplementary file 1 — PDF-Japanese [file fmj-7-122-s001.pdf]

要旨

目的: 磁気センサ型指タッピング装置（UB-2）を用いて，指タップ運動を実施し，認知機能の重症度をよく反映する手指運動を，指運動の定量化パラメータを用いて重回帰分析を行った.

方法: アルツハイマー病（Alzheimer’s disease: AD）と診断された患者 44 名（平均年齢 73.8±7.0 歳），軽度認知障害（Mild Cognitive Impairment: MCI）と診断された患者 20 名（平均年齢 76.7±4.2 歳）の合計 64 名を対象とした．指タップ運動の課題は， 片手（左右）タップ動作，両手同時，両手交互を実施した．計測後，算出した手指のパラメータから Mini-Mental State Examination （MMSE）を予測するために，年齢，性別で調整した重回帰分析を実施した.

結果: 標準化偏回帰係数は，オープニング速度ピーク時の距離比率の標準偏差，接触時間の標準偏差等のパラメータで高かった．決定係数（coefficient of determination: R^2）は 0.1～0.28 であった.

結論: これらのパラメータが認知機能と有意に関連するパラメータである可能性が示唆された．今後，認知症患者の早期診断ツールとしての可能性を検討していくために，大規模集団の高齢者を対象に計測を行っていきたい.

キーワード: 認知機能 指タップ 巧緻性 MMSE

序論

世界保健機関は, 2015 年に世界中で 4,700 万人の認知症が存在すると推定し，人口の高齢化により 2050 年までに 3 倍になると予測した. [1] 日本でも，認知症患者数は増加しており，現在約 630 万人の認知症患者が存在している．さらに約 400 万人と推計されている軽度認知障害（Mild Cognitive impairment: MCI）をあわせると, 65 歳以上高齢者の約 4 人に 1 人が認知症，または予備軍といわれており大きな社会問題となっている. [2]

ランセット認知症予防，介入，ケアに関する国際委員会（Lancet International Commission on Dementia Prevention, Intervention and Care）は，認知症予防の重要性を指摘しており，認知症発症が 5 年遅れた場合，認知症の有病率は半減する可能性があると報告した. [3] 現段階では，認知症の病理学的な低下を抑えることはできないが，発症予防や進行予防を試みていくためにも，認知症のリスクを早期に検出することが重要である.

認知症全体の約 60%を占めるアルツハイマー病（Alzheimer’s disease: AD）は，初期症状としてエピソード記憶障害を認め，他の認知機能障害を伴いながら慢性進行性の経過をたどる疾患である.[4,5] 一方, MCI は正常な認知と認知症の中間状態として定義されており, AD の前臨床段階に関連している.[6] 最近では, MCI を含む認知症の初期段階や発症前から，記憶障害のみならず運動障害 [7-9] や感覚障害 [10,11] を認めるといった報告がなされてきており，脳の機能的な側面を総合的に検討していくことが重要である．私たちはその 1 つとして手指の運動（指タップ運動）に着目し，はっきりとした認知症の初期症状が現れてくる前の段階で，脳の病理学的な変化に伴う何らかの手指の微細な異常を呈しているのではないかと仮説を立てた．その仮説を実証するために, 認知症患者の運動障害を簡易に短時間で評価できる方法として，手指の運動機能計測装置（磁気センサ型指タッピング装置: UB-1 日立コンピュータ機器株式会社）を用いた認知症患者の指タップ運動に対する予備的研究を進めた. [12] 結果，認知症の初期段階である MCI の時期から手指運動巧緻性の低下を認める可能性や，認知機能が重症化するほど低下を認める手指のパラメータを検出した．認知症患者の手指機能に関する研究では，微細な運動コントロールや手指巧緻性の低下，指タップ回数が減少するといった報告がある. [13-15] また, Muller[16] は軽度の AD 患者について，Ott[17] らは軽度から中等度の AD 患者の指タップ速度が低下すると報告している.

手指巧緻性に関する研究では，巧緻性が年齢とともに低下していくという報告や性別による違いも指摘されている. [18-20] 上述したように，これまで認知症患者の手

指機能に関する検討はなされているが，認知症患者の認知機能と手指機能の関連を多変量解析で検討した報告はない.

よって本研究の目的は，前述の磁気センサ型指タッピング装置の改良型であるUB-2（Figure 1）を用いて指タップ運動を実施し，認知機能の重症度をよく反映する手指運動を，指運動の定量化パラメータを用いて重回帰分析を行うことである.

方法

対象

当センターのもの忘れセンターで, AD，もしくはMCIと診断された患者を対象とした．今回MCIを含めたのは，認知機能の重症度を反映する手指運動の予測精度を高めるために幅広く選定した. ADの診断基準は, National Institute on Aging-Alzheimer's Association (NIA/AA)による診断基準[21]に従い, MCIはPetersenの診断基準[22]に従った. 対象者は, AD患者49名（平均年齢73.4±6.7）, MCI患者20名（平均年齢76.7±4.2）の合計69名であった．除外基準は，意識障害，失語症や失行症などの高次脳機能障害,脳卒中などで明らかな麻痺や感覚障害，手指巧緻性障害，振戦を呈する者とした.

倫理的配慮

全ての対象者またはその家族に対して，事前に口頭及び書面にて本実験の趣旨を十分に説明し，同意を得られた者とした．なお，本研究は当センターの倫理･利益相反委員会で承認を得ている（承認番号: 623-7）.

問診票の記入と認知機能評価

AD，およびMCI患者に対して，指タップ計測前に構造化インタビューにて，氏名や年齢，性別，既往歴，利き手，認知症に関わる背景情報を取得するための確認を問診票にて行った（Table 1）.また，一般的に認知機能の重症度を定量的に評価可能なMini-Mental State Examination（MMSE）を実施した.

計測方法

使用機器は，磁気センサ型指タッピング装置（UB-2 マクセル株式会社）を用いた（Figure 1）．装置は，サイズW69×D28×H140.5mm，重量210g，内蔵電池満充電時の連続使用時間は約 3.5 時間である．計測は，静かな環境で椅子座位にて実施した．計測方法は, Figure 1の黄色のケーブルを左手，赤色のケーブルを右手に装着する．それ

それの母指と示指の背側（爪側）に装着し，指タップ運動（2 指の繰り返し開閉動作）を行う．指タップ運動の課題は，片手（左手のみ，右手のみ）でのタップ動作，両手同時（左右同時のタップ動作），両手交互（左右交互のタップ動作）の 4 課題を行った（Figure 2）．計測時の注意点としては，①肘関節は机に接地させず，②前腕中間位で脇を締め，③手関節は軽度背屈位，④中指～小指を軽く握った姿勢で計測を行った（Figure 3）．また，各動作計測前に，タップ動作の理解度を確認するため, 5 秒程度の練習を 1 回行い，「できるだけ速く行ってください」と教示した．練習後に各課題動作を左手，右手，両手同時動作，両手交互動作の順で行い, 15 秒間計測した．

なお，本研究において 4 課題を選定した理由については，①利き手と非利き手の差異が大きいこと，②両手の計測については，両手の従属性（両手同時）と独立性（両手交互）の評価が重要であると考えているからである．

統計方法

手指のパラメータから MMSE を予測するために，年齢，性別で調整した重回帰分析を実施した．なお，重回帰分析に用いるパラメータは，ステップワイズ変数選択法によって定めた．初期モデルは定数頁のみとし，変数を投入する基準となる F 値は 0.05，除外する基準は 0.10 とした．付属ソフトウェア Just Tap からは指タップ計測後, 44 個のパラメータが算出されるが,[12] 本研究では，重回帰分析の適用において問題となる多重共線性を回避するため，類似した意味をもつパラメータを割愛して，Table 2 に示す 36 個のパラメータを用いた．統計的有意水準は $p<0.05$ とし，解析ソフトは SPSS Statistics ver. 26.0 を使用した．

結果

除外基準に基づいて，認知症群 64 名（AD 患者 44 名, MCI 患者 20 名）が対象となった．対象者は全て右利きであった. AD 患者では，脳卒中の既往があった 1 名，頚椎症の既往があった 2 名，大脳皮質基底核変性症と診断されていた 1 名，振戦があり日常生活に支障を認めた 1 名の合計 5 名を除外した．対象者の特性を Table 3 に示す．

高齢者と AD 患者の波形の 1 例

測定波形の 1 例を Figure 4 に示す．（A）は患者の付き添いで来院された家族で，社会生活が自立しており，もの忘れ外来の受診歴がない 70 歳代の男性高齢者の測定

結果である．（B）は本研究の解析の対象に含んでいる 70 歳代の AD 女性患者の測定結果である．（A）では，タップ数が多く，タップ動作も安定していた．一方（B）では，タップ数も少なく，タップ動作も一定せずばらつきを認めた．

従属変数に MMSE を用いた重回帰分析

MMSE を従属変数とし，年齢と性別で調整した重回帰分析の結果を Table 4 に示す．従属変数に対する独立変数の相対的な関連の強さを示す「標準化偏回帰係数」は，オープニング速度ピーク時の距離比率の標準偏差（Table 2 No.17），接触時間の標準偏差（Table 2 No.25），タップインターバルの標準偏差（Table 2 No.29），オープニング速度ピーク時の距離比率の平均（Table 2 No.14），距離の極大点の近似直線の傾き（Table 2 No.5）の順に大きかった．その他のパラメータに関しては有意差を認めなかった．また，各課題の関係性について，片手タップ（右手）と両手同時の右手では，オープニング速度ピーク時の距離比率の標準偏差（Table 2 No.17），片手タップ（左手）と両手同時の左手では，接触時間の標準偏差（Table 2 No.25），両手交互の右手と左手では，タップインターバルの標準偏差（Table 2 No.29）で標準化偏回帰係数が 0.3 以上（もしくは-0.3 以下）であった．決定係数（coefficient of determination: R^2）は 0.1 〜0.28 であった．

考察

本研究では，手指のパラメータから MMSE を予測するために，年齢，性別で調整した重回帰分析を実施した．パラメータ別では，主にオープニング速度ピーク時の距離比率の標準偏差（No.17）や接触時間の標準偏差（No.25）で標準化偏回帰係数が高い傾向を示した．認知症患者の MMSE と手指機能との関連については，接触時間のパラメータが有用であるといった報告があり，[23] 本研究でも接触時間に関して標準偏回帰係数が高い傾向を示したことから，接触時間に関するパラメータは，認知症患者の認知機能と有意に関連するパラメータの 1 つである可能性が高いと考える．また，オープニング速度ピーク時の距離比率の標準偏差(No.17)の標準偏回帰係数が高い傾向を示したことから，MMSE の点数が低い者は，最大速度になる距離が一定ではないために，手指の開き方が安定していない可能性が示唆された．よって本研究の結果より，接触時間の標準偏差(No.25)は手指の接触時のばらつきの評価を，オープニング速度ピーク時の距離比率の標準偏差(No.17)は手指の非接触時のばらつきを評価する指標として有用なパラメータである可能性が高いと考える．

また，各課題の関係性については，片手タップ（右手）と両手同時の右手では，オープニング速度ピーク時の距離比率の標準偏差（No.17）で有意差を認め，片手タップ（左手）と両手同時の左手では，接触時間の標準偏差（No.25）で有意差を認めた．両手交互の右手と左手は，タップインターバルの標準偏差（No.29）で有意差を認めた．先行研究では，片手タップ（左手）と両手同時の左手の接触時間が，健常高齢者よりも AD や MCI 患者で有意に長い結果が得られたといった報告があり,[23] 先行研究を支持する結果となった．また，認知症患者は健常高齢者よりも，両手交互のような両手の協調性やリズムを必要とする課題で手指機能低下を認めるといった報告もあり,[12] 本研究でも両手交互において，リズムに関するパラメータであるタップインターバルの標準偏差(No.29)で有意差を認めた．認知症患者では，大脳皮質の萎縮に加えて両手の協調性に関与する大脳基底核[24]や脳梁[25]の萎縮を認めるといわれている．両手交互は，片手や両手同時に比較して，両手を独立して動かす必要があり最も難易度が高いため，手指機能に差を認めやすいと考える．

MCI から AD への移行期間に関しては，年間 4〜10%前後といわれ,[26]MCI の 50%が 5 年間で AD へ移行する[27]といった報告があるが，いまだ移行期間に関する一定の結論に至っていない．最近では，脳脊髄液中の Aβ や総タウ，リン酸化タウなどのバイオマーカー[28,29]や MRI での脳の形態異常の出現,[30]SPECT[31]および PET[32]における脳血流の異常分布をもとにある程度の早期診断が可能になってきた．しかし，これらの高度技術を元にした画像診断などは，対象者の経済的・身体的負担や，計測や分析に時間を要すという課題がある．それらに比べ指タップ計測は，(1)検査までの導入が容易であること，(2)手指の限定した運動であるため，認知機能障害を呈していても計測が可能なこと，(3)計測が短時間（5 分程度）であるため対象者への負担が少ないと考えられる．今後，認知症患者の手指機能について研究を進めることで，指タップ計測から認知症患者の認知機能をある程度推定することができれば，早期診断の一助となる可能性がある．

本研究の限界として，以下の点が挙げられる．1 つ目に巧緻性に影響を与える要因として，潜在的に併存する錐体外路障害があるが,[33,34]今回，錐体外路障害の影響について評価していないことが挙げられる．2 つ目に認知機能の程度を MMSE のみで判断していることが挙げられる．3 つ目に利き手の判断には，Edinburgh 利き手テスト[35]等を用いることが一般的であるが，今回，被検者の疲労を考慮し問診票のみで利き手を判断してしまったことが挙げられる．今後，MMSE 単独の検査ではなく，注意・遂行機能に関する評価も実施し，認知機能を総合的に把握していく必要性や，利き手テス

トを用いた利き手の判定を行っていく必要がある.

今回, 磁気センサ型指タッピング装置を用いて指タップ運動を実施し, 認知機能の重症度をよく反映する手指運動を, 指運動の定量化パラメータを用いて検討を行った. 結果, 指運動の定量化パラメータを使用することによって, オープニング速度ピーク時の距離比率の標準偏差（No.17）と接触時間の標準偏差(No.25)が認知機能と有意に関連するパラメータであることが示唆された. 今後, この指タップ動作を認知症のスクリーニング評価の1つとして使用することを目標としており, 地域の健康教室など大規模集団の高齢者を対象に計測を行っていきたい. 片手（左右）, 両手同時, 両手交互の4課題を実施するのではなく, 1つの課題に選定することが可能となれば, 認知症の運動障害を簡易にかつ短時間で検出することが可能となると考えている.

利益相反

共著者の佐野佑子と神鳥明彦は株式会社日立製作所の従業員であり, 水口寛彦はマクセル株式会社の従業員である. 国立長寿医療研究センターは, 株式会社日立製作所, およびマクセル株式会社と共同研究を実施している. 本研究で使用された装置は, マクセル株式会社から国立長寿医療研究センターに貸与されたものである.

謝辞

本研究は, JSPS 科研費 JP19K11339 の助成を受けた.

References

1. Alzheimer's Disease International. Policy Brief for Heads of Government: The Global Impact of Dementia 2013-2050. Alzheimer's Disease International 2013.
2. Cabinet Office, government of Japan. Kourei shakai hakusho (Annual Report on the Aging Society); 2016. (in Japanese). <https://www8.cao.go.jp/kourei/whitepaper/w-2016/html/zenbun/s1_2_3.html>. (Accessed February 21, 2020).
3. Livingston G, Sommerlad A, Orgeta V, et al. Dementia prevention, intervention, and care. Lancet 2017; 16: 2673-734.
4. Souza LC, Sarazin M, Goetz C, Dubois B. Clinical investigations in primary care. Front Neurol Neurosci 2009; 24: 1-11.
5. Dubois B, Picard G, Sarazin M. Early detection of Alzheimer's disease: new diagnostic criteria. Dialogues Clin Neurosci 2009; 11: 135-9.
6. Langa KM, Levine DA. The diagnosis and management of mild cognitive impairment: a clinical review. JAMA 2014; 312: 2551-61.
7. Sabia S, Dugravot A, Dartigues JF, Abell J, Elbaz A, Kivimäki M, Singh-Manoux A. Physical activity, cognitive decline, and risk of dementia: 28 year follow-up of Whitehall II cohort study. BMJ 2017; 357: j2709.
8. Carment L, Abdellatif A, Lafuente-Lafuente C, Pariel S, Maier MA, Belmin J, Lindberg PG. Manual Dexterity and Aging: A Pilot Study Disentangling Sensorimotor From Cognitive Decline. Front Neurol 2018; 9: 910.
9. Jeon SY, Han SJ, Jeong JH, Fregni F. Effect of exercise on balance in persons with mild cognitive impairment. NeuroRehabilitation 2014; 35: 271-8.
10. Kouzuki M, Suzuki T, Nagano M, Nakamura S, Katsumata Y, Takamura A, Urakami K. Comparison of olfactory and gustatory disorders in Alzheimer's disease. Neurol Sci 2018; 39: 321-8.
11. Roalf DR, Moberg MJ, Turetsky BI, Brennan L, Kabadi S, Wolk DA, Moberg PJ. A quantitative meta-analysis of olfactory dysfunction in mild cognitive impairment. J Neurol Neurosurg Psychiatry 2017; 88: 226-32.
12. Suzumura S, Osawa A, Nagahama T, Kondo I, Sano Y, Kandori A. Assessment of finger motor skills in individuals with mild cognitive impairment and patients with Alzheimer's disease: relationship between finger-to-thumb tapping and cognitive function. Jpn J Compr Rehabil Sci 2016; 7: 19-28.

13. Kluger A, Gianutsos JG, Golomb J, Ferris SH, George AE, Franssen E, Reisberg B. Patterns of motor impairement in normal aging, mild cognitive decline, and early Alzheimer's disease. J Gerontol B Psychol Sci Soc Sci 1997; 52B: 28-39.
14. Tomita Y, Tanaka S, Takahashi S, Takeuchi N. Detecting cognitive decline in community-dwelling older adults using simple cognitive and motor performance tests. Geriatr Gerontol Int 2020; 20: 212-7.
15. Yan JH, Dick MB. Practice effects on motor control in healthy seniors and patients with mild cognitive impairment and Alzheimer's disease. Neuropsychol Dev Cogn B Aging Neuropsychol Cogn 2006; 13: 385-410.
16. Muller G, Weisbrod S, Klingberg F. Finger tapping frequency and accuracy are decreased in early stage primary degenerative dementia. Dementia 1991; 2: 169-72.
17. Ott BR, Ellias SA, Lannon MC. Quantitative assessment of movement in Alzheimer's disease. J Geriatr Psychiatry Neurol 1995; 8: 71-5.
18. Ranganathan VK, Siemionow V, Sahgal V, Yue GH. Effects of aging on hand function. J Am Geriatr Soc 2001; 49: 1478-84.
19. Dayanidhi S, Valero-Cuevas FJ. Dexterous manipulation is poorer at older ages and is dissociated from decline of hand strength. J Gerontol A Biol Sci Med Sci 2014; 69: 1139-45.
20. Lawrence EL, Fassola I, Werner I, Leclercq C, Valero-Cuevas FJ. Quantification of dexterity as the dynamical regulation of instabilities: comparisons across gender, age, and disease. Front Neurol 2014; 5: 53.
21. Mckhann GM, Knopman DS, Chertkow H, et al. The diagnosis of dementia due to alzheimer’s disease: Recommendations from the national Institute on aging-alzheimer’s association workgroups on diagnostic guidelines for alzheimer’s disease. Alzheimers Dement 2011; 7: 263-9.
22. Petersen RC, Smith GE, Waring SC, Ivnik RJ, Tangalos EG, Kokmen E. Mild cognitive impairment: clinical characterization and outcome. Arch Neurol 1999; 56: 303-8.
23. Suzumura S, Osawa A, Maeda N, Sano Y, Kandori A, Mizuguchi T, Yin Y, Kondo I. Differences among patients with Alzheimer's disease, older adults with mild cognitive impairment and healthy older adults in finger dexterity. Geriatr Gerontol Int 2018; 18: 907-14.
24. Cho H, Kim JH, Kim C, et al. Shape changes of the basal ganglia and thalamus in

Alzheimer's disease: a three-year longitudinal study. J Alzheimers Dis 2014; 40: 285-95.
25. Zhu M, Wang X, Gao W, Shi C, Ge H, Shen H, Lin Z. Corpus callosum atrophy and cognitive decline in early Alzheimer's disease: longitudinal MRI study. Dement Geriatr Cogn Disord 2014; 37: 214-22.
26. Davis M, O Connell T, Johnson S, Cline S, Merikle E, Martenyi F, Simpson K. Estimating Alzheimer's Disease Progression Rates from Normal Cognition Through Mild Cognitive Impairment and Stages of Dementia. Curr Alzheimer Res 2018; 15: 777-88.
27. Gauthier S, Reisberg B, Zaudig M, et al. Mild cognitive impairment. Lancet 2006; 367: 1262-70.
28. Agarwal R, Tripathi CB. Diagnostic Utility of CSF Tau and Aβ (42) in Dementia: A Meta-Analysis. Int J Alzheimers Dis 2011; 2011: 503293.
29. Ahmed RM, Paterson RW, Warren JD, Zetterberg H, O'Brien JT, Fox NC, Halliday GM, Schott JM. Biomarkers in dementia: clinical utility and new directions. J Neurol Neurosurg Psychiatry 2014; 85: 1426-34.
30. Frisoni GB, Fox NC, Jack CR Jr, Scheltens P, Thompson PM. The clinical use of structural MRI in Alzheimer disease. Nat Rev Neurol 2010; 6: 67-77.
31. Yeo JM, Lim X, Khan Z, Pal S. Systematic review of the diagnostic utility of SPECT imaging in dementia. Eur Arch Psychiatry Clin Neurosci 2013; 263: 539-52.
32. Pike KE, Ellis KA, Villemagne VL, Good N, Chételat G, Ames D, Szoeke C, Laws SM, Verdile G, Martins RN, Masters CL, Rowe CC. Cognition and beta-amyloid in preclinical Alzheimer's disease: data from the AIBL study. Neuropsychologia 2011; 49: 2384-90.
33. Tosto G, Monsell SE, Hawes SE, Mayeux R. Pattern of extrapyramidal signs in Alzheimer's disease. J Neurol 2015; 262: 2548-56.
34. Portet F, Scarmeas N, Cosentino S, Helzner EP, Stern Y. Extrapyramidal signs before and after diagnosis of incident Alzheimer disease in a prospective population study. Arch Neurol 2009; 66: 1120-6.
35. Oldfield RC. The assessment and analysis of handedness: The Edinburgh inventory. Neuropsychologia 1971; 9: 97-113.

Figure 1 磁気センサ型指タッピング装置 (UB-2)

黄色のケーブルは, 左手の親指と人差し指に取り付け, 赤いケーブルは右手の親指と人差し指に取り付ける. 機器本体は PC と接続され, PC 上で指タップ時の波形を確認することが可能. UB-1 よりも軽量で持ち運びが容易である.

Figure 2 指タップ運動

(1) 非利き手タップ: 左母指と示指で可能な限り速く指タップ運動を行う

(2) 利き手タップ: 右母指と示指で可能な限り速くタップ運動を行う

(3) 両手同時タップ: 両手を同じタイミングで可能な限り速く指タップ運動を行う

(4) 両手交互タップ: 左右交互に可能な限り速く指タップ運動を行う

上記(1)-(4)は, 2 指間の距離を 3～4cm に保ち, 15 秒間実施する

Figure 3 計測時の注意点

①: 中指から小指は軽く握った状態

②: 手関節は軽度背屈位

③: 前腕中間位で脇を締める

④: 肘関節は机に接地させない

Figure 4 高齢者と AD 患者の波形の 1 例

（A）70 歳代男性高齢者　（B）70 歳代女性 AD 患者
